# Oxidative Stress and Periodontal Disease in Obesity

**DOI:** 10.1097/MD.0000000000003136

**Published:** 2016-03-25

**Authors:** Erhan Dursun, Ferda Alev Akalın, Tolga Genc, Nese Cinar, Ozcan Erel, Bulent Okan Yildiz

**Affiliations:** From the Department of Periodontology (ED, FAA, TG), Faculty of Dentistry; Division of Endocrinology and Metabolism (NC, BOY), Department of Internal Medicine, Faculty of Medicine, Hacettepe University; and Department of Biochemistry (OE), Faculty of Medicine, Yıldırım Beyazıt University, Ankara, Turkey.

## Abstract

Periodontal disease is a chronic inflammatory disease of the jaws and is more prevalent in obesity. Local and systemic oxidative stress may be an early link between periodontal disease and obesity. The primary aim of this study was to detect whether increased periodontal disease susceptibility in obese individuals is associated with local and systemic oxidative stress. Accordingly; we analyzed periodontal status and systemic (serum) and local (gingival crevicular fluid [GCF]) oxidative status markers in young obese women in comparison with age-matched lean women.

Twenty obese and 20 lean women participated. Periodontal condition was determined by clinical periodontal indices including probing depth, clinical attachment level, gingival index, gingival bleeding index, and plaque index. Anthropometric, hormonal, and metabolic measurements were also performed. Blood and GCF sampling was performed at the same time after an overnight fasting. Serum and GCF total antioxidant capacity (TAOC), and total oxidant status (TOS) levels were determined, and oxidative stress index (OSI) was calculated.

Clinical periodontal analyses showed higher gingival index and gingival bleeding index in the obese group (*P* = 0.001 for both) with no significant difference in probing depth, clinical attachment level, and plaque index between the obese and the lean women. Oxidant status analyses revealed lower GCF and serum TAOC, and higher GCF and serum OSI values in the obese women (*P* < 0.05 for all). GCF TOS was higher in the obese women (*P* < 0.05), whereas there was a nonsignificant trend for higher serum TOS in obese women (*P* = 0.074). GCF TAOC values showed a negative correlation with body mass index, whereas GCF OSI was positively correlated with fasting insulin and low-density lipoprotein-cholesterol levels (*P* < 0.05 for all). Clinical periodontal indices showed significant correlations with body mass index, insulin, and lipid levels, and also oxidant status markers.

Our results suggest that young obese, otherwise healthy, women show findings of early periodontal disease (gingival inflammation) compared with age-matched healthy lean women, and that local/periodontal oxidative stress generated by obesity seems to be associated with periodontal disease.

## INTRODUCTION

Obesity is a major public health problem worldwide, affecting both developed and developing countries.^1^ Studies have shown that overweight and obesity are associated with several chronic diseases including periodontal disease.^1^ Periodontal disease is also one of the most common chronic inflammatory diseases affecting the world population, which is initiated by colonization of pathogenic bacteria followed by exaggerated inflammatory response resulting in alveolar bone loss.^2^ Periodontal disease defines pathological inflammatory conditions of the gingival and tooth-supporting structures that may result in partial or complete loss of teeth. Gingivitis and chronic periodontitis are the most common forms. Gingivitis is simply defined as the inflammation of the gingival with no signs of bone destruction. Periodontitis is described as clinical and radiographic signs of destruction of tooth-supporting structures. The main cause for periodontal diseases is the microbial dental plaque. Several studies suggested that periodontal disease-associated bacteria could also penetrate gingival tissues, enter the bloodstream, and potentially induce transient bacteremia after chewing, tooth brushing, and periodontal treatment.^3,4^

Although periodontitis is initiated by subgingival plaque biofilm, the larger part of tissue breakdown seems to be mediated by abnormal host response to specific bacteria and their products.^5,6^ The abnormal response is characterized by exaggerated inflammation involving the release of proteolytic enzymes and reactive oxygen species (ROS).^2^ Numerous studies established the association between periodontal disease and systemic disorders, including obesity, metabolic syndrome, diabetes, cardiovascular disease, and nonalcoholic fatty liver disease.^7–10^

Obesity as a chronic inflammatory disease is defined as a state of oxidative stress.^11^ Increased levels of proinflammatory cytokines such as tumor necrosis factor-α, interleukin-6, and interleukin-1β, and decreased adiponectin levels in obesity are responsible for the overproduction of reactive oxygen and nitrogen species by macrophages and monocytes, leading to increased oxidative stress.^11^ Obesity has a modified inflammatory background and a hyperoxidative state, leading to a higher susceptibility to bacterial infection, which may facilitate the beginning and/or progression of periodontitis. Oxidative stress may be a meeting point between 2 inflammatory diseases such as obesity and periodontal disease.^6^

Oxidative stress is a condition caused by a harmful increase in the production of ROS, emerging when there is an imbalance between ROS levels and host antioxidant defenses, resulting in DNA, lipid, and protein damage.^6^ One of the defense mechanisms of neutrophils against bacteria is the release of ROS.^2^ An increase in ROS production, or a decrease in antioxidant defense, may result in oxidative stress, which can cause significant damage in all tissues of the human body.^2^ Oxidative stress has been implicated in many disease states in humans, including periodontitis,^12^ and is one of the main factors studied to explain the pathophysiological mechanisms of inflammatory conditions, such as obesity and periodontitis.^13,14^

Oxidative stress has been variably determined by the measurement of a decrease in total antioxidant capacity (TAOC), or more often, by estimation of the products of oxidative damage to lipids, proteins, and DNA.^15^ Recently, the association between obesity and periodontal disease is attracting attention and has become one of the current issues in both medical and dental research. Normally, there is a fragile balance between ROS production and tissue concentrations of antioxidants in the body. Measurements of TAOC and total oxidant status (TOS) in serum and gingival crevicular fluid (GCF) may elucidate the local and systemic interaction between obesity and periodontal disease.^16^ GCF is a complex mixture of substances derived from serum, leukocytes, and structural cells of periodontium and oral bacteria.^17^ GCF is the most appropriate fluid for sampling when investigating local periodontal status due to its ability to pass through the tissues, and accumulates the biomarkers of local tissue events.^6^ GCF analysis has been an object of investigations for early detection of periodontitis.^18^ Data from GCF analyses support the conclusion that local antioxidant scavenging defenses are compromised in periodontal inflammation.^6^ Although many biomarkers are used to evaluate oxidative stress, as the measurement of different oxidant molecules is not practical and their oxidant effects are additive, measurement of TAOC and TOS in serum and GCF seems to be reliable methods and can provide a new and practical approach to determine the oxidative status in periodontal disease.^13,16^

The null hypothesis tested in the present study is periodontal inflammation and local and/or systemic oxidative stress increased in young obese woman in terms of clinical periodontal and oxidative stress indices as primary outcome measures against alternative hypothesis, as no difference is present between young obese and lean women. The aim of this study was to analyze the effect of obesity on both local and systemic TAOC and TOS levels and oxidative stress index (OSI) values and to determine periodontal status in young obese women compared with age-matched lean women.

## METHODS

### Study Groups

The present study included a total of 40 individuals, comprising 20 obese women (mean age 27.29 ± 4.89 years) and 20 age and sex-matched lean controls (mean age 26.11 ± 4.09 years). The obese group included women who presented to the Endocrinology Outpatient Clinic at Hacettepe University consecutively and who met the entry criteria. The control group included healthy lean women who volunteered to participate in the study.

The inclusion criteria were having no systemic disease; having no periodontal treatment history; no antibiotics, anti-inflammatory, or any other drug use during the past 3 months; being never-smoker,; and no alcohol or antioxidant vitamin tablet use. The study was approved by the Institutional Review Board of Hacettepe University (Decision number: FON 07/13). The participants were informed about the study protocol, and all of them signed the written informed consent.

Anthropometric measurements including height, weight, body mass index (BMI), waist circumference, and waist-to-hip ratio (WHR) (waist [midway between the lower rib margin and the iliac crest]; hip [widest circumference over the great trochanters]) were determined. Clinical periodontal examinations were performed on all subjects and the periodontal parameter values were recorded. Two days after the periodontal examinations, GCF samples were obtained from the participants. After these procedures, the individuals underwent a standard 120 minutes of 75 g oral glucose tolerance test (OGTT) between 08.00 and 10.00 am, fasting blood samples were taken, and 120-minute glucose levels were obtained. For blood and GCF samplings, participants had fasted overnight. All blood and GCF sampling procedures were performed on the same day.

### Periodontal Examinations

The periodontal condition of all participants was determined by measuring probing depth (PD) and clinical attachment level (CAL) by using Williams periodontal probe (Michigan O probe with Williams markings). PD is the distance between the gingival margin and the base of the sulcus/pocket. It may change due to periodontal disease, and/or changes in the position of the gingival margin. Higher scores of PD indicate the periodontal destruction or gingival overgrowth. CAL is the distance between the base of the pocket and a fixed point on the tooth crown such as the cemento-enamel junction. Changes in the CAL can be due only to gain or loss of attachment (the connection between tooth-root and periodontal bone). Increased PD and loss of clinical attachment are pathognomonic for periodontal disease. Therefore, pocket probing is a crucial and mandatory procedure in diagnosing periodontitis. Clinical periodontal parameters including gingival index (GI),^19^ gingival bleeding index (GBI),^20^ and plaque index (PI)^21^ were also recorded. GI provides an assessment of gingival inflammatory status. Higher scores of GI indicate higher gingival inflammation—0: normal to healthy gingiva; 1: mild inflammation: slight change in color and slight edema; 2: moderate inflammation: redness, edema, and glazing; 3: severe inflammation: marked redness, edema, and ulceration. PI refers to the amount of dental/bacterial plaque—0: no plaque in gingival area; 1: no plaque visible by the unaided eye, but plaque is made visible on the point of the probe after it has been moved across surface at entrance of gingival crevice; 2: gingival area is covered with a thin to moderately thick layer of plaque, deposit is visible to the naked eye; 3: heavy accumulation of soft matter, interdental area is stuffed with soft debris. Panoramic radiographs were used to determine the level of periodontal bone for all included individuals. Probing depth, CAL, and GBI were measured in duplicate at 6 sites (mesial, distal, and median points at the buccal and palatal aspects), whereas GI and PI were measured in duplicate at 4 sites (mesiobuccal, midbuccal, distobuccal, and midpalatal).

### Collection of Serum and GCF Samples

All samples were collected in the morning after overnight fasting. The participants were instructed not to drink and eat anything in the morning, and were questioned about their protocol adherence before sample collection. To avoid possible irritation and stimulation of GCF flow during clinical measurements, samples were obtained 2 days after the clinical measurements and between 8:00 and 10:00 in the morning. The samples were obtained according to the method described by Rudin et al^18^ using standardized paper strips from maxillary anterior and premolar teeth. No attempt was made to specifically select sites with deep pockets, samples were pooled per subject to ensure sufficient assay sensitivity, and the patient was used as the unit of analyses. Site-specific differences were therefore not analyzed. Twelve GCF samples were collected from each participant. The samples were collected from mesiobuccal, mid, distobuccal, and palatal sites on maxillary premolars and incisors (at least 3 teeth). Paper strips were placed at the entrance of tooth crevice, and were inserted to a standardized depth of 1 mm. Sampling time was also standardized as 30 seconds. Papers with visible blood contaminations were discarded. To eliminate the risk of evaporation, paper strips with GCF were immediately transported to previously calibrated Periotron 8000 for volume determination. The strips with GCF were placed in sterile, firmly wrapped Eppendorf tubes immediately and stored at −80°C until the day of laboratory analysis.

Venous blood for serum was collected in plain tubes that were initially kept at room temperature for 30 minutes. After blood collection, the tubes were kept at 4°C for 30 minutes before centrifugation at 1000×*g* for 10 minutes at room temperature. Serum samples were aliquoted into cryogenic vials and stored in liquid nitrogen.

### Laboratory Studies

#### Hormone and Blood Glucose Assays

Plasma glucose levels were measured by the glucose oxidase technique (Roche Molecular Biochemicals, Mannheim, Germany). Insulin levels were measured by RIA (Diagnostic Systems Laboratories, Inc., Webster, TX). The intra and interassay coefficient of variations (CVs) were 4.5% and 8.9%, respectively. Plasma total cholesterol (TC), high-density lipoprotein (HDL), low-density lipoprotein LDL, and triglyceride (TG) levels were determined by enzymatic colorimetric method (Roche Molecular Biochemicals, Mannheim, Germany). The average intra and interassay CVs were 1.4% and 2.2%, respectively.

#### TAOC Assay

Serum and GCF TAOC levels were determined using a commercially available assay kit.^22^ This method is based on the bleaching of the characteristic color of a more stable 2,29-azino-bis (3-ethylbenz-thiazoline-6-sulfonic acid) radical cation by antioxidants. The results were expressed in millimoles 6-hydroxy-2,5,7, 8-tetramethylchroman-2-carboxylic acid equivalents per liter.

#### TOS Assay

Serum and GCF TOS levels were determined using a commercially available assay kit.^16^ This method is based on the oxidation of ferrous ion to ferric ion in the presence of various oxidative species in acidic medium and the measurement of the ferric ion by xylenol orange. The results were expressed in micromoles of H_2_O_2_ per liter. TOS method is colorimetric and automated, and the precision of this assay is excellent at <3%.

#### OSI Values

The percentage ratio of TOS/TAOC was used to calculate the OSI. To perform the calculation, the TAOC, given in millimoles trolox equivalent per liter, was converted to micromoles equivalent per liter, and the OSI value was calculated using the following formula for serum and GCF separately.

OSI = ([TOS {mmol/L}]/[TAOC {mmol} Trolox equivalent]/l × 100).^23^

#### Outcome Variables

Periodontal disease that was expressed by clinical periodontal indices and panoramic radiographs and oxidative stress that was expressed by TOS and TAOC levels have been assumed as outcome variables to detect the effect of obesity on susceptibility of periodontal disease through local and systemic oxidative stress, which is one of the most favorite candidates of periodontal disease onset and progression.^2^

#### Exposure Variable

Obesity has been assumed as an exposure variable, and we used the World Health Organization (WHO)-defined BMI-based categories of underweight (BMI <18.5 kg/m^2^), normal weight (BMI = 18.5–24.9 kg/m^2^), overweight (BMI = 25.0–29.9 kg/m^2^), and obese (BMI ≥ 30 kg/m^2^) to determine the study groups as lean (normal weight [BMI = 18.5–24.9 kg/m^2^]) and obese (BMI ≥ 30 kg/m^2^).

### Statistical Analyses

A statistical software package (SPSS 20) was used for all statistical analyses. The normality of the data distribution was tested by Shapiro–Wilk test. For normally distributed data, comparisons among 2 groups were performed by Student *t* test. For non-normally distributed data, Mann–Whitney *U* test was used. Pearson/Spearman coefficient was used to examine the significant correlations. The results were reported as mean ± SD. Statistical significance between groups according to associated parameters was defined as *P* < 0.05. A priori power analysis using a G∗Power version 3.1.9 statistical software program indicated that, with 13 subjects in each group, the study would have >80% power to detect significant difference at a level of 0.05 between the 2 groups according to GCF TAOC values. After the completion of the study, considering the SD of each group, the power values were confirmed to be >80%.

## RESULTS

### Anthropometric, Hormonal, and Metabolic Parameters

Anthropometric, hormonal, and metabolic features of individuals are given in Table 1. Obese women compared with the lean group had significantly higher values for BMI, WHR, glucose-120, fasting insulin, insulin-120, and lower HDL levels (*P* < 0.05 for all). No statistically significant differences were found between the groups for TC, TG, and LDL levels (*P* > 0.05 for all).

**TABLE 1:**
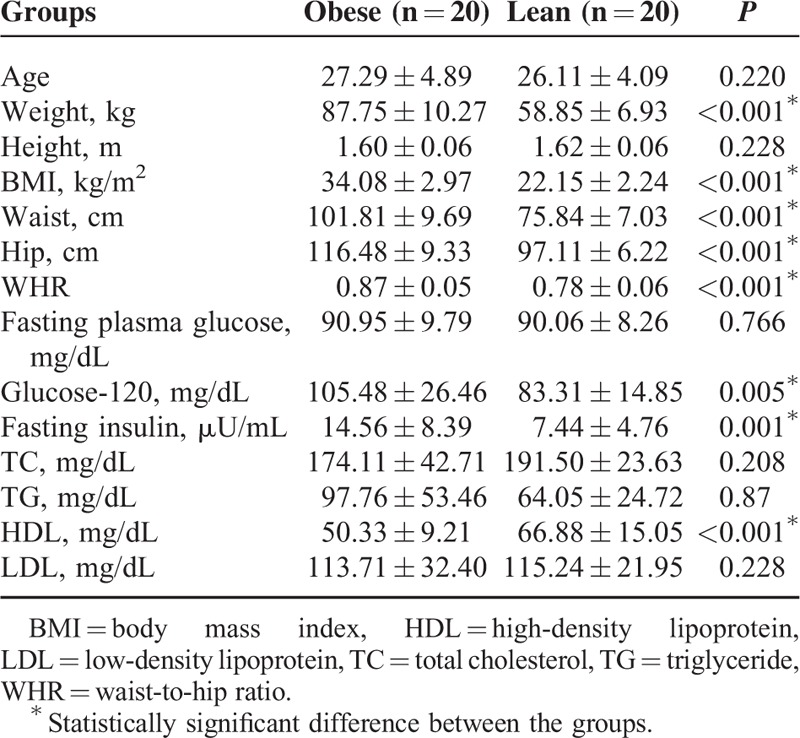
Anthropometric, Hormonal, and Metabolic Parameters

### Clinical Periodontal Parameters

Table 2 summarizes the clinical periodontal parameters of the study groups. No significant differences were found between the groups according to PD, CAL, and PI values (*P* > 0.05), whereas GI and GBI were significantly higher in the obese group (*P* = 0.001). No proximal bone loss, deep periodontal pocket (>3 mm), and periodontitis were detected in any included individual according to clinical periodontal and radiographic examinations.

**TABLE 2:**
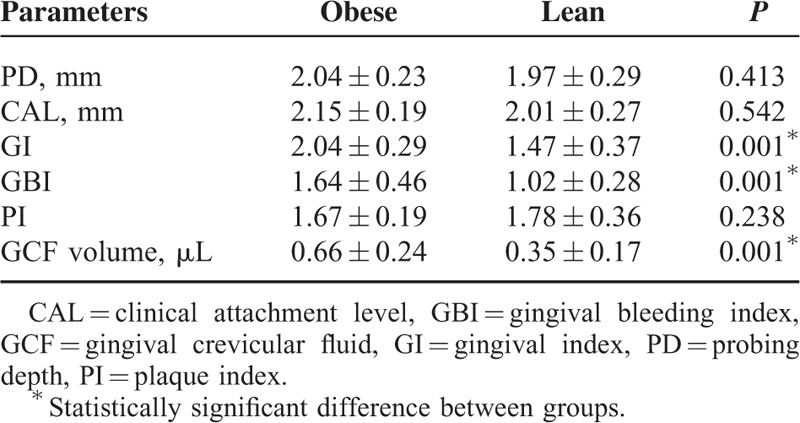
Clinical Periodontal Parameters

### TAOC and TOS Levels

Table 3 summarizes the oxidative status in serum and GCF of the participants. Serum TAOC was significantly lower in the obese group (*P* = 0.032), whereas no significant differences were found between the groups according to serum TOS levels (*P* = 0.074). Regarding GCF oxidant status, TAOC was significantly lower, whereas TOS level was significantly higher in the obese groups compared with lean controls (*P* = 0.038 and 0.042, respectively).

**TABLE 3:**
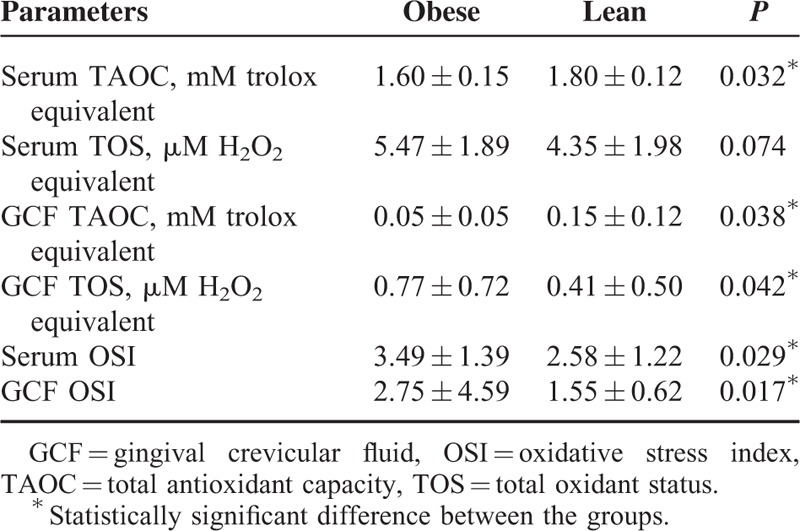
Oxidative Status Parameters

### OSI Values

When comparing the OSI values between the groups; serum and GCF OSI values were significantly higher in the obese group than the lean control group (*P* = 0.029 and 0.017, respectively).

### Correlation Analyses

Table 4 summarizes the significant correlations between oxidant status, and anthropometric and hormonal parameters. In all individuals, serum TAOC was significantly negatively correlated with serum TOS, BMI values and fasting glucose, glucose-120, fasting insulin, and TG levels (*P* < 0.05 for all) There was a significant positive correlation between serum TOS and TG levels (*r* 0.387; *P* < 0.05). When evaluating the relationship between local/periodontal oxidant status, and anthropometric and hormonal measurements, significant negative correlation was found between GCF TAOC and BMI (*r* −0.389; *P* < 0.05), whereas significant positive correlation was found between GCF TOS and LDL levels (*r* 0.451; *P* < 0.05). GCF TOS also showed significant positive correlations with CAL and GI scores (*r* 0.309, 0.460, respectively; *P* < 0.05 for both). Gingival index and GBI, which are clinical indicators of local/periodontal inflammation, were significantly positively correlated with BMI, WHR, and fasting insulin levels, and negatively correlated with HDL levels (*P* < 0.05 for all). PD was also significantly positively correlated with fasting plasma glucose level (*r* 0.384; *P* < 0.05). When OSI values were evaluated in all individuals, there were significant positive correlations between serum OSI values, and serum TG and fasting insulin levels, whereas GCF OSI values showed significant positive correlations with serum LDL, fasting insulin levels, and GI and GBI scores (*P* < 0.05 for all).

**TABLE 4:**
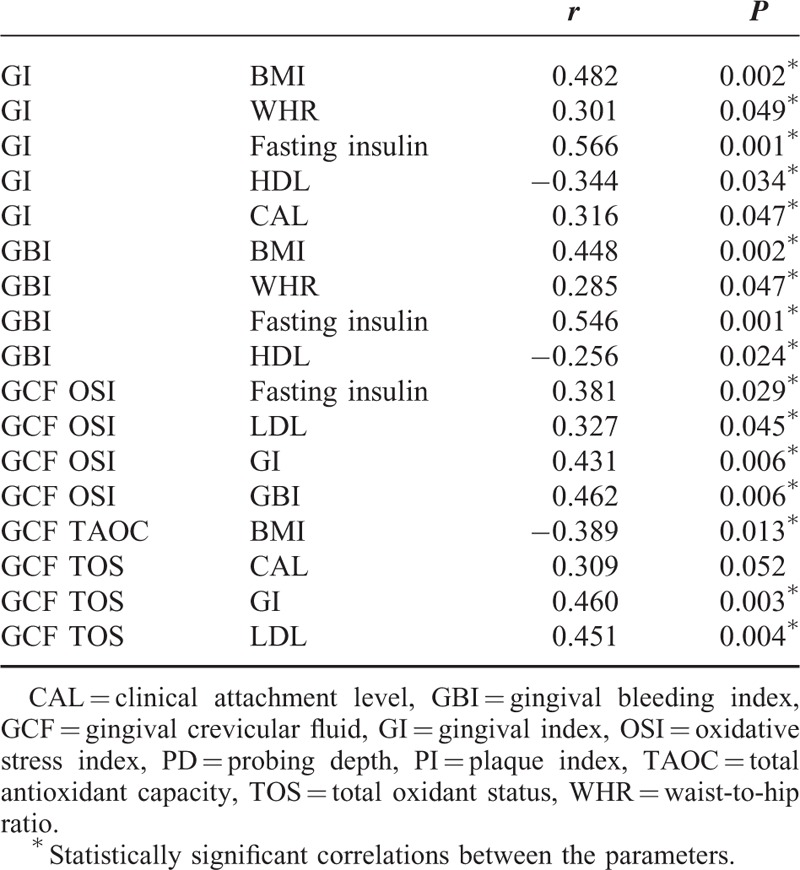
Correlations Between Oxidative Status Markers, Clinical Periodontal Parameters and Anthropometric, Hormonal, and Metabolic Parameters

## DISCUSSION

To the best of our knowledge, this is the first study investigating the influence of obesity on systemic (serum) and local (GCF) TAOC, and total oxidative status, and the association of these parameters with periodontal state in humans. Our findings indicate significantly lower TAOC levels and significantly higher TOS and OSI levels in serum and GCF in the obese group compared with the lean control group. When clinical periodontal situations of obese and lean individuals were compared, there was no significant difference in PD or CAL, whereas higher gingival inflammation and bleeding were found in obese individuals despite similar plaque levels. It may be considered that prominent gingival inflammation at this young age may establish a ground for future chronic periodontitis in these obese women.^24^

Studies have shown that obesity is one of the risk factors for the development of periodontal tissue destruction.^25^ Higher prevalence of periodontitis in obese women has been reported in the literature. Dalla Vecchia et al^26^ reported that obese women had an 80% greater risk of presenting periodontitis than their lean counterparts. Our finding of higher scores of GI and GBI in obese but otherwise healthy and young individuals, compared with age and sex-matched lean controls, denotes that periodontal health is deteriorated and gingival inflammation (gingivitis) is a common finding in these women. In the present study, the higher GCF volume further supports this finding. Our radiographic analyses revealed no periodontal bone loss in these subjects, indicating that they had the periodontal disease at the gingival level (gingivitis), but not periodontal bone loss level (periodontitis), which might be due to the relatively young age of the group.

It has been widely accepted that oxidative stress plays an important role in the pathologic mechanisms of different forms of periodontitis.^27,28^ In a meta-analysis including 16 studies from different countries reported oxidative stress biomarkers, serum TAOC levels were lower and serum NO levels were higher in patients with chronic periodontitis compared with healthy subjects, suggesting that periodontal inflammation may trigger systemic oxidative stress.^12^ Studies have shown that periodontitis is associated with decreased TAOC and increased TOS levels.^13,29^ Enhanced oxidative stress is evident in obesity like other diverse diseases including cancer, diabetes, neurodegenerative diseases, and atherosclerosis. Tomofuji et al^30^ found higher gingival oxidative stress in obese rats and they reported that obesity may induce gingival oxidative damage and accentuate gingival inflammation with increasing blood ROS. The present findings suggest that obese individuals have a local/periodontal pro-oxidative state and decreased systemic antioxidant capacity. Our data indicate that obese individuals have increased oxidative stress both systemically and locally, even though they are young, nondiabetic, and nonsmoker. When considering that chronic periodontitis mainly affects the adult population >35 years of age, local OSI may be an early marker of the development of periodontal disease. Oxidative stress analysis may be a valuable tool to understand the common pathologic mechanisms between 2 inflammatory conditions such as obesity and periodontal disease.

We determined meaningful correlations among GI and GBI scores, and GCF TAOC and TOS levels and GCF OSI values. Local oxidative stress was found to be associated with gingival inflammation parameters as expected. The anthropometric and hormonal parameters such as BMI, weight, and waist measurements, fasting insulin, and glucose levels were also found to be positively correlated with GI and GBI scores. TOS in GCF seems to be associated with serum LDL levels, whereas gingival inflammation seems to be negatively correlated with HDL levels. We have shown that there were significant correlations between GCF and serum OS markers, and adiposity, lipid, glucose, and insulin values. These correlations suggest that systemic oxidative stress is associated with BMI, insulin resistance, and dyslipidemia, as expected. Additionally, according to correlation analyses, abdominal adiposity and metabolic alterations such as insulin resistance in obese young women might influence the risk of periodontal disease through local and systemic oxidative stress even before development of clinical diabetes or cardiovascular disease.

There are some limitations caused by heterogeneity of the studies, such as design, sample size, and the methods of the biomarker measurements. There is a remarkable variability among different studies regarding the types of oxidative stress biomarkers.^31^ The great variability among different studies may also be caused by different degrees of the periodontal disease (chronic periodontitis), different study populations (sex and age), and different biological specimens (serum, plasma, GCF, or whole blood), and also by the absence of a well-defined certain biomarker of oxidative stress.^31^ Although specific biomarkers should be examined to clearly identify oxidative stress, assessment of oxidative stress through the evaluation of the TAOC and TOS may be rational to elucidate the oxidant status.^16,22,27,29^

An important limitation in some of the studies about possible relationship between obesity and periodontitis is the presence of diabetes, which is knowingly associated with the generation of oxidative free radicals.^14^ In the present study, no diabetic patient was included and no significant differences were found between the groups according to fasting glucose levels. Obese individuals are subject to the manifestation of diabetes, because adipose tissue secretes proinflammatory cytokines that are the main mediators of insulin resistance.^14,31^ In accordance with this relationship, higher fasting insulin and glucose-120 levels, which are insulin resistance markers, were found in obese individuals in the present study. Smoking is a well-known contributor of ROS and a reducer of antioxidants.^32^ Furthermore, smoking is one of the strongest risk factors for periodontitis.^33^ Haffajee and Socransky^34^ also reported that overweight or obese women have a higher risk of periodontitis than normal BMI after adjustment of age and smoking. The present study was controlled for smoking, and all included individuals were females; thus smoking and sex were excluded as confounding factors that might have an impact on the results of the study.

Oral cavity as a part of body also may have systemic effects. Pathogens found in periodontal infection are strictly linked to other organs, the main of which is spleen.^10^ Periodontopathogen bacteria such as *Porphyromonas gingivalis*, *Treponema denticola*, and *Tannerella forsythia* are frequently detected in periodontal sites during chronic periodontal inflammation. In a recent study, mice chronically infected with major periodontal pathogens demonstrated consistent miR-146a expression both locally in the maxilla and systemically in the spleen.^10^ These localized and systemic expressions suggest that miR-146a expression might be due to a bacterial presence and/or inflammation in periodontium and spleen, also based upon the finding that genomic DNA from *P gingivalis*, *T denticola*, or *T forsythia* was detected in oral plaque samples, liver, and spleen.^10^ Obese individuals have higher incidence of infection, suggesting impaired immune function. It has been reported that spleen as an important regulation center of the body's immune-metabolic-endocrine network plays an important role in obesity, with its essential effects on chronic infection and immune response.^35^

The limitations of our study are a relatively small sample size and cross-sectional design (precludes cause–effect relationship). Evaluating the effect of microbial plaque samples and bacteria compounds would have also increased the value of the study. Additionally, vitamin D levels, which have been reported as a protective factor for periodontal health, may be a potential confounding factor and counted as a limitation for the present study.^36^ Future longitudinal investigations on the interrelationships between obesity, periodontal disease, and oxidative stress are necessary to increase the knowledge in this field.

## CONCLUSIONS

Our results suggest that the susceptibility to periodontal disease may significantly increase, and systemic and local/periodontal oxidant status seems to be affected in obesity. Reduced antioxidant capacity and generation of oxidative stress may be a pathophysiological link for higher susceptibility to periodontitis in obesity.

## References

[1] Yusuf S Hawken S Ounpuu S Obesity and the risk of myocardial infarction in 27,000 participants from 52 countries: a case-control study. *Lancet* 2005; 366:1640–1649. 16271645 10.1016/S0140-6736(05)67663-5

[2] Chapple IL Matthews JB The role of reactive oxygen and antioxidant species in periodontal tissue destruction. *Periodontol 2000* 2007; 43:160–232. 17214840 10.1111/j.1600-0757.2006.00178.x

[3] Forner L Larsen T Kilian M Incidence of bacteremia after chewing, tooth brushing and scaling in individuals with periodontal inflammation. *J Clin Periodontol* 2006; 33:401–407. 16677328 10.1111/j.1600-051X.2006.00924.x

[4] Kinane DF Riggio MP Walker KF Bacteraemia following periodontal procedures. *J Clin Periodontol* 2005; 32:708–713. 15966875 10.1111/j.1600-051X.2005.00741.x

[5] Page RC Kornman KS The pathogenesis of human periodontitis: an introduction. *Periodontology 2000* 1997; 14:9–11. 9567963 10.1111/j.1600-0757.1997.tb00189.x

[6] Waddington RJ Moseley R Embery G Reactive oxygen species: a potential role in the pathogenesis of periodontal diseases. *Oral Dis* 2000; 6:138–151. 10822357 10.1111/j.1601-0825.2000.tb00325.x

[7] Kinane D Bouchard P Periodontal diseases and health: consensus report of the Sixth European Workshop on Periodontology. *J Clin Periodontol* 2008; 35:333–337. 18724860 10.1111/j.1600-051X.2008.01278.x

[8] Yoneda M Naka S Nakano K Involvement of a periodontal pathogen, *Porphyromonas gingivalis* on the pathogenesis of non-alcoholic fatty liver disease. *BMC Gastroenterol* 2012; 12:16. 22340817 10.1186/1471-230X-12-16 PMC3305584

[9] Tarantino G Should nonalcoholic fatty liver disease be regarded as a hepatic illness only? *World J Gastroenterol* 2007; 13:4669–4672. 17729388 10.3748/wjg.v13.i35.4669 PMC4611188

[10] Nahid MA Rivera M Lucas A Polymicrobial infection with periodontal pathogens specifically enhances microRNA miR-146a in ApoE-/- mice during experimental periodontal disease. *Infect Immun* 2011; 79:1597–1605. 21263019 10.1128/IAI.01062-10 PMC3067556

[11] Furukawa S Fujita T Shimabukuro M Increased oxidative stress in obesity and its impact on metabolic syndrome. *J Clin Invest* 2004; 114:1752–1761. 15599400 10.1172/JCI21625 PMC535065

[12] Liu Z Liu Y Song Y Systemic oxidative stress biomarkers in chronic periodontitis: a meta-analysis. *Dis Markers* 2014; Article ID: 931083. 10.1155/2014/931083 PMC4247950 25477703

[13] Akalin FA Baltacioglu E Alver A Lipid peroxidation levels and total oxidant status in serum, saliva and gingival crevicular fluid in patients with chronic periodontitis. *J Clin Periodontol* 2007; 34:558–565. 17555410 10.1111/j.1600-051X.2007.01091.x

[14] Bullon P Morillo JM Ramirez-Tortosa MC Metabolic syndrome and periodontitis: is oxidative stress a common link? *J Dent Res* 2009; 88:503–518. 19587154 10.1177/0022034509337479

[15] Wei PF Ho KY Ho YP The investigation of glutathione peroxidase, lactoferrin, myeloperoxidase and interleukin-1beta in gingival crevicular fluid: implications for oxidative stress in human periodontal diseases. *J Periodontal Res* 2004; 39:287–293. 15324348 10.1111/j.1600-0765.2004.00744.x

[16] Erel O A new automated colorimetric method for measuring total oxidant status. *Clin Biochemistry* 2005; 38:1103–1111. 10.1016/j.clinbiochem.2005.08.008 16214125

[17] Uitto VJ Gingival crevice fluid: an introduction. *Periodontol 2000* 2003; 31:9–11. 12656992 10.1034/j.1600-0757.2003.03101.x

[18] Rudin HJ Overdiek HF Rateitschak KH Correlation between sulcus fluid rate and clinical and histological inflammation of the marginal gingiva. *Helvet Odontol Acta* 1970; 14:21–26. 5438796

[19] Loe H The gingival index, the plaque index and the retention index systems. *J Periodontol* 1967; 38:610–616. 5237684 10.1902/jop.1967.38.6.610

[20] Muhlemann HR Son S Gingival sulcus bleeding: a leading symptom in initial gingivitis. *Helvet Odontol Acta* 1971; 15:107–113. 5315729

[21] Silness J Loe H Periodontal disease in pregnancy. II. Correlation between oral hygiene and periodontal condtion. *Acta Odontol Scandinav* 1964; 22:121–135. 14158464 10.3109/00016356408993968

[22] Erel O A novel automated method to measure total antioxidant response against potent free radical reactions. *Clin Biochem* 2004; 37:112–119. 14725941 10.1016/j.clinbiochem.2003.10.014

[23] Kosecik M Erel O Sevinc E Increased oxidative stress in children exposed to passive smoking. *Int J Cardiol* 2005; 100:61–64. 15820286 10.1016/j.ijcard.2004.05.069

[24] Schatzle M Loe H Burgin W Clinical course of chronic periodontitis. I. Role of gingivitis. *J Clin Periodontol* 2003; 30:887–901. 14710769 10.1034/j.1600-051x.2003.00414.x

[25] Wood N Johnson RB Streckfus CF Comparison of body composition and periodontal disease using nutritional assessment techniques: Third National Health and Nutrition Examination Survey (NHANES III). *J Clin Periodontol* 2003; 30:321–327. 12694430 10.1034/j.1600-051x.2003.00353.x

[26] Dalla Vecchia CF Susin C Rosing RV Overweight and obesity as risk indicators for periodontitis in adults. *J Periodontol* 2005; 76:1721–1728. 16253094 10.1902/jop.2005.76.10.1721

[27] Baltacioglu E Yuva P Aydin G Lipid peroxidation levels and total oxidant/antioxidant status in serum and saliva from patients with chronic and aggressive periodontitis. Oxidative stress index: a new biomarker for periodontal disease? *J Periodontol* 2014; 85:1432–1441. 24635543 10.1902/jop.2014.130654

[28] D’Aiuto F Parkar M Nibali L Periodontal infections cause changes in traditional and novel cardiovascular risk factors: results from a randomized controlled clinical trial. *Am Heart J* 2006; 151:977–984. 16644317 10.1016/j.ahj.2005.06.018

[29] Chapple IL Milward MR Dietrich T The prevalence of inflammatory periodontitis is negatively associated with serum antioxidant concentrations. *J Nutr* 2007; 137:657–664. 17311956 10.1093/jn/137.3.657

[30] Tomofuji T Yamamoto T Tamaki N Effects of obesity on gingival oxidative stress in a rat model. *J Periodontol* 2009; 80:1324–1329. 19656033 10.1902/jop.2009.080621

[31] Boesing F Patino JS da Silva VR The interface between obesity and periodontitis with emphasis on oxidative stress and inflammatory response. *Obes Rev* 2009; 10:290–297. 19207875 10.1111/j.1467-789X.2008.00555.x

[32] Pryor WA Stone K Oxidants in cigarette smoke. Radicals, hydrogen peroxide, peroxynitrate, and peroxynitrite. *Ann N Y Acad Sci* 1993; 686:12–27. 8512242 10.1111/j.1749-6632.1993.tb39148.x

[33] Haber J Wattles J Crowley M Evidence for cigarette smoking as a major risk factor for periodontitis. *J Periodontol* 1993; 64:16–23. 8426285 10.1902/jop.1993.64.1.16

[34] Haffajee AD Socransky SS Relation of body mass index, periodontitis and *Tannerella forsythia*. *J Clin Periodontol* 2009; 36:89–99. 19207883 10.1111/j.1600-051X.2008.01356.x

[35] Tarantino G Spleen: A new role for an old player. *World J Gastroenterol* 2011; 17:3776–3784. 21987619 10.3748/wjg.v17.i33.3776 PMC3181438

[36] Zhan Y Samietz S Holtfreter B Prospective study of serum 25-hydroxy vitamin D and tooth loss. *J Dent Res* 2014; 93:639–644. 24828383 10.1177/0022034514534985 PMC4293729

